# HES6-1 and HES6-2 Function through Different Mechanisms during Neuronal Differentiation

**DOI:** 10.1371/journal.pone.0015459

**Published:** 2010-12-02

**Authors:** Filipe Vilas-Boas, Domingos Henrique

**Affiliations:** Instituto de Medicina Molecular, Faculdade de Medicina de Lisboa, Lisboa, Portugal; National Institutes of Health, United States of America

## Abstract

**Background:**

Notch signalling plays a central role in the mechanisms regulating neuronal differentiation in the vertebrate nervous system. The transcriptional repressors encoded by *Hes* genes are the main effectors of this pathway, acting in neural progenitors during the lateral inhibition process to repress proneural genes and inhibit differentiation. However, *Hes6* genes seem to behave differently: they are expressed in differentiating neurons and facilitate the activity of proneural genes in promoting neurogenesis. Still, the molecular mechanisms underlying this unique function of *Hes6* genes are not yet understood.

**Methodology/Principal Findings:**

Here, we identify two subgroups of *Hes6* genes that seem conserved in most vertebrate species and characterize a novel *Hes6* gene in chicken: c*Hes6-1*. The embryonic expression pattern of c*Hes6-1* suggests roles for this gene in the formation of the pancreas, nervous system and in the generation of body asymmetry. We show that c*Hes6-1* is negatively regulated by Notch signalling in the developing embryonic spinal cord and in pancreatic progenitors, but requires Notch for the observed asymmetric expression at the lateral mesoderm. Functional studies by ectopic expression in the chick embryonic neural tube revealed that cHES6-1 up-regulates the expression of c*Delta1* and c*Hes5* genes, in contrast with overexpression of cHES6-2, which represses the same genes. We show that this activity of cHES6-2 is dependent on its capacity to bind DNA and repress transcription, while cHES6-1 seems to function by sequestering other HES proteins and inhibit their activity as transcriptional repressors.

**Conclusions/Significance:**

Our results indicate that the two chick HES6 proteins act at different phases of neuronal differentiation, contributing to the progression of neurogenesis by different mechanisms: while cHES6-2 represses the transcription of *Hes* genes, cHES6-1 acts later, sequestering HES proteins. Together, the two cHES6 proteins progressively shut down the Notch-mediated progenitor program and ensure that neuronal differentiation can proceed.

## Introduction

The vertebrate central nervous system derives from an embryonic structure called the neural tube. In this tissue, dividing neural progenitors reside in the ventricular zone (VZ), near the lumen of the tube. Progenitor cells have attachments at the apical and basal sides of the neuroepithelium and their nuclei show a characteristic interkinetic nuclear movement, with mitotic nuclei being always located apically ([1]; reviewed in [2]). After division, neuroepithelial cells either remain as progenitors in the VZ or commit to differentiation, moving out of the VZ to a more basal region called the mantle layer, where neuronal differentiation proceeds. During neurogenesis, there is a balance between progenitor proliferation and differentiation, maintaining a resident population of progenitors to ensure that neurogenesis can progress and produce the correct number (and types) of neuronal cells during development. This balance is regulated by Notch signalling (reviewed in [3]), a pathway that is based on cell-cell interactions: after the contact between a membrane bound ligand (Delta or Serrate) of one cell and a membrane bound Notch receptor on another cell, the receptor suffers a proteolytic cleavage catalyzed by γ-secretase, releasing the intracellular domain (NICD) from the membrane. The NICD protein then translocates to the nucleus where it binds CSL (CBF1, Suppressor of Hairless, Lag-1) and Mastermind (MAM), turning CSL from a transcriptional repressor into a transcriptional activator. The tripartite NICD/CSL/MAM complex activates the transcription of several downstream targets, the best characterized being the *Hes* genes (*Drosophila Hairy and Enhancer of Split* homologues), which encode basic helix-loop-helix (bHLH) transcriptional repressors (reviewed in [4]). HES proteins contain a basic region for DNA binding and a helix-loop-helix region for homo- or heterodimerization, as well as an Orange domain involved in protein-protein interactions specificity and a C-terminal tetrapeptide (WRPW) for interactions with the co-repressor Groucho/TLE (reviewed in [5], [6]). HES proteins normally act as dimers, bind to specific sequences named N- or E- boxes in target promoters, recruit the co-repressor TLE and repress the transcription of target genes. Known targets include the proneural genes, which are involved in promoting neuronal differentiation. During neurogenesis, newborn neurons express high levels of proneural factors, which promote expression of the ligands Delta or Serrate. These ligands signal to neighbouring Notch-expressing progenitors, where Notch activation leads to HES protein expression and a block on the activity of proneural genes, thereby preventing progenitor differentiation, in a process called lateral inhibition (reviewed in [4], [6], [7]).

Although vertebrate HES proteins usually function as Notch effectors to inhibit neuronal differentiation in the developing nervous system, studies in mouse, *Xenopus* and *in vitro* have shown that one particular HES protein, HES6, acts differently, promoting neurogenesis when ectopically expressed [8]–[12]. This seems to be due to HES6's ability to inhibit the activity of anti-neurogenic HES proteins, leading to an increase on the expression and activity of proneural proteins, such as Neurogenins, in cells where they are already expressed [8]–[11], [13]. In turn, Neurogenins increase HES6 levels in a positive feedback-loop [8], thus reinforcing the activity of the proneural differentiation cascade. Another proneural protein, ASCL1, was shown to bind to E boxes in the m*Hes6* promoter [14], [15], and might also be a positive regulator of mHES6 during neurogenesis.

Multiple mechanisms have been shown to contribute to the inhibitory activity of HES6 on anti-neurogenic HES factors, but these do not seem to involve a DNA-binding dependent transcriptional activity of HES6. Instead, the mouse HES6 protein was shown to form heterodimers with HES1 and prevent recruitment of the transcriptional co-repressor TLE, necessary for HES1 repressive activity [9], [10], [13]. In addition, binding of HES6 to HES1 seems to cause proteolytic degradation of the heterodimer, mediated by phosphorylation of a specific serine residue in HES6 [10], [13].

In the chick embryo, two *Hes6* genes are expressed and the product of one of them, cHES6-2, has been shown to cooperate with the proneural proteins to promote neuronal differentiation [16], although the underlying molecular mechanisms are not known. The function of the second HES6 protein, cHES6-1 [16], is also still unknown. In this study, we investigated the expression and function of cHES6-1 and show that it is expressed in all three germ layers of the chick embryo, particularly in the nervous system, heart, and pancreas. c*Hes6-1* is asymmetrically expressed in the mesoderm lateral to the primitive streak, suggesting a role for this gene in the formation of body asymmetry. c*Hes6-1* expression is negatively regulated by Notch signalling in the embryonic spinal cord and pancreas. However, in the mesoderm lateral to the primitive streak, c*Hes6-1* expression requires Notch activity, suggesting different regulation of this gene in distinct tissues. In the embryonic spinal cord, the two chick *Hes6* genes are transiently expressed in differentiating neurons and function in the genetic cascade regulating neuronal differentiation, acting by different mechanisms. While cHES6-2 functions as a transcriptional repressor, cHES6-1 functions by sequestering and inhibiting other HES proteins. Together, the two chick HES6 proteins regulate neurogenesis by contributing to release differentiating neurons from Notch signalling.

## Results

### HES6 proteins are divided in two subgroups

**Figure 1:**
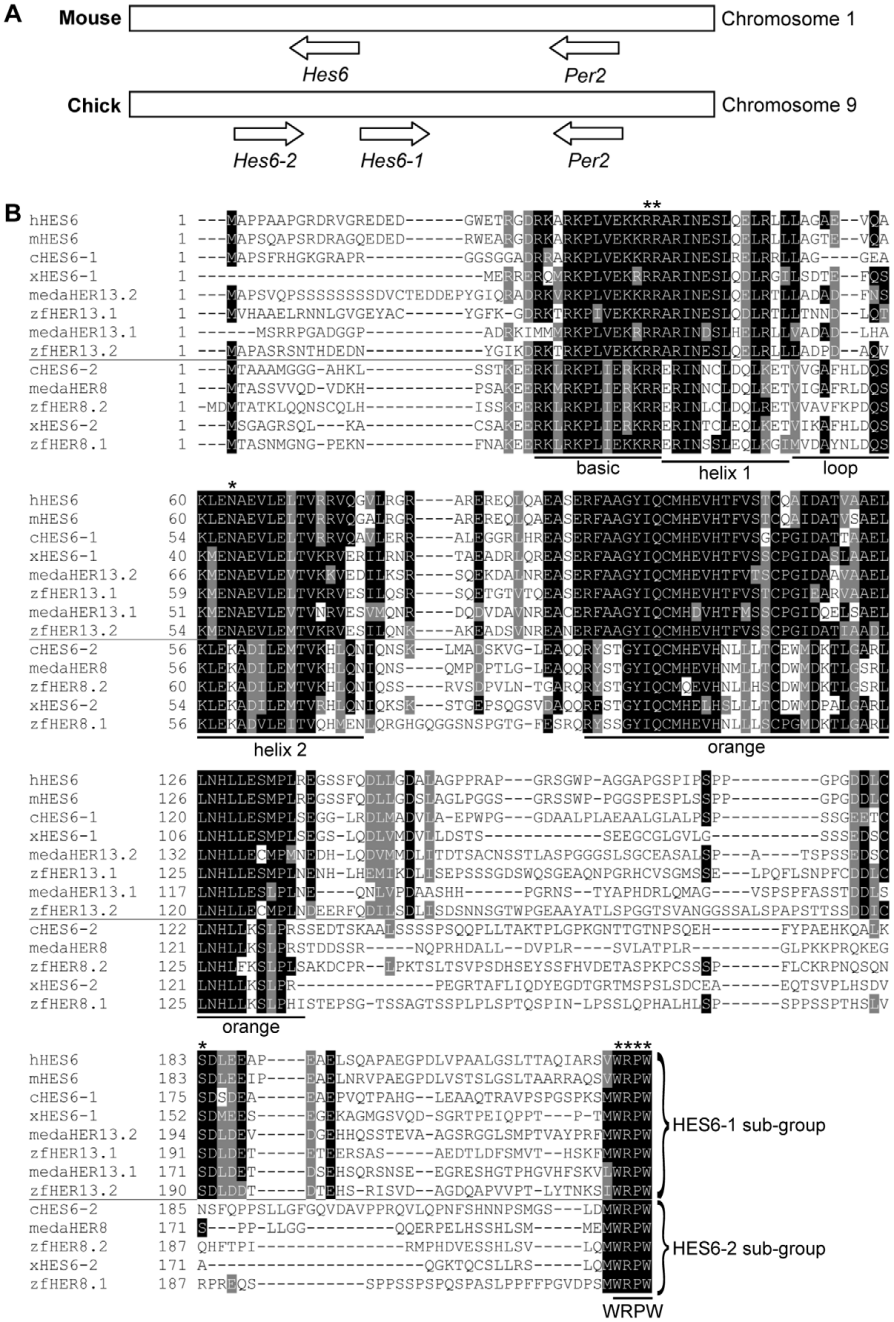
HES6 subgroups. (A) Location and orientation of the single mouse *Hes6* and the two chick *Hes6* genes in chromosomes 1 and 9, respectively. In both genomes, *Hes6* genes are present in close proximity to the *Period2* gene. (B) Sequence alignments of HES6 proteins from different species. HES6 protein sequences were obtained in Ensemble or NCBI and have the following accession numbers. *Homo sapiens*: HES6, ENSP00000272937. *Mus musculus*: HES6, ENSMUSP00000084062. *Gallus gallus*: HES6-2, ENSGALP00000008850. *Xenopus tropicalis:* HES6-1, ENSXETP00000013003; HES6-2, NP_001072210. *Oryzias latipes*: HER13.1, ENSORLP00000020797; HER13.2, ENSORLP00000019320; HER8, ENSORLP00000019320. *Danio rerio*: HER13.1, ENSDARP00000012990; HER13.2 ENSDARP00000021078; HER8.1, ENSDARP00000010206; HER8.2, ENSDARP00000103093. Accession number of *Gallus gallus* HES6-1 is unavailable, but can be identified in GenBank (BI393243). Shaded areas represent regions of homology. The HES6 proteins are divided in two subgroups: HES6-1 and HES6-2. The main protein domains are indicated. Amino acid residues that were mutated in our experiments are marked with asterisks. h: human; m: mouse; c: chick; x: *Xenopus*; meda: medaka; zf: zebrafish.

We have previously reported the existence of two *Hes6*-like genes in the chick genome, c*Hes6-1* and c*Hes6-2* [16]. A more detailed analysis of available genomic data reveals that the two genes are linked in chromosome 9, close to the *Period2* gene (Ensemble ref. ENSGALG00000005521) (Fig. 1A). We found that two different *Hes6* genes are equally present in the genomes of zebrafish, medaka and *Xenopus*, while only one *Hes6* gene is present in mouse and humans. Alignment of the various HES6 proteins reveals that they can be classified into two distinct subgroups, with unique structural features (Fig. 1B). These include a particular serine residue specifically present in all HES6-1 proteins (position 175 of cHES6-1), reported to be important for mHES6-dependent induction of neurogenesis [10], and a lysine residue present in the bHLH domain of all HES6-2 proteins (position 59 of cHES6-2), suggested to be essential for transcriptional repression ability of HES proteins [17]. However, the major structural difference between the two subgroups of HES6 proteins concerns the shorter loop in the bHLH domain of the HES6-1 proteins, when compared with HES6-2 subgroup.

### c*Hes6-1* expression pattern

**Figure 2:**
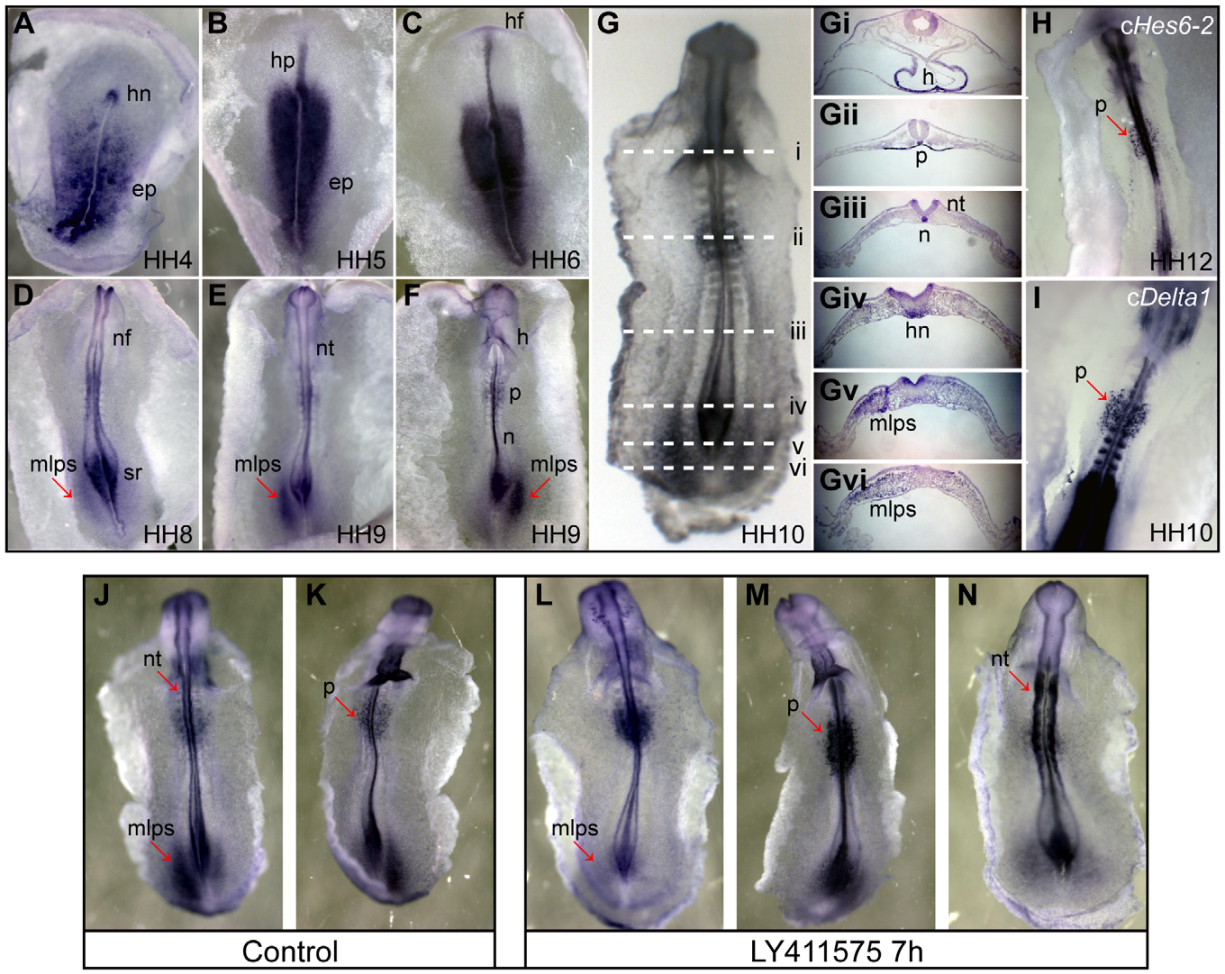
c*Hes6-1* expression pattern and response to Notch inhibition. (A-G) Expression of c*Hes6-1* at stages (A) HH4, (B) HH5, (C) HH6, (D) HH8, (E) HH9 (dorsal view), (F) HH9 (ventral view), (G) HH10. (Gi-Gvi) Sections through the regions marked with the white dashed lines on HH10 embryo in (G). Arrows in (D-F) point to the asymmetric expression of c*Hes6-1* in the mesoderm lateral to the primitive streak. (H-I) Ventral view of embryos showing expression of (H) c*Hes6-2* at HH12 and (I) c*Delta1* at HH10 in pancreatic progenitors, identified by arrows. (J-N) Expression of c*Hes6-1* in embryos treated with the Notch signalling inhibitor LY411575. Down-regulation of c*Hes6-1* can be detected in the mesoderm lateral to the primitive streak (L, dorsal view) and up-regulation of c*Hes6-1* in pancreatic progenitors (M, ventral view) and neural tube (N, dorsal view), when compared to control embryos treated with PBS (J, dorsal, and K, ventral view). Arrows in (J-N) point to regions of the embryo where c*Hes6-1* expression if affected by Notch signalling inhibition. ep: epiblast; h: heart; hf: head fold; hn: Hensen's node; hp: head process; mlps: mesoderm lateral to the primitive streak; n: notochord; nf: neural fold; nt: neural tube; p: pancreatic progenitors; sr: sinus rhomboidalis.

The expression pattern of c*Hes6-1* throughout chick embryonic development was determined by *in situ* hybridization on whole-mount embryos and cryostat sections. At HH4 (Hamburger and Hamilton stages [18]), c*Hes6-1* is expressed in the epiblast and Hensen's node (Fig. 2A). As the node starts regressing at HH5, expression of c*Hes6-1* is also detected in the emerging head process (Fig. 2B). During gastrulation, c*Hes6-1* is always expressed around Hensen's node and in the forming notochord. Other sites of c*Hes6-1* expression in early embryos (HH5-12) include the neural folds and neural tube, the cranial placodes, the infundibulum and the prospective heart (Fig. 2C-Gi,Giii,Giv and data now shown). Expression can also be detected in the lateral mesoderm flanking the regressing node in 5 somite embryos, but only in the left side (arrow in Fig. 2D). This asymmetric expression continues throughout HH9 and HH10 and finally equalizes in both sides at HH11 (Fig. 2E-G,Gv,Gvi and data not shown). At 7 somites, expression of c*Hes6-1* is also detected in a salt-and-pepper pattern in the endoderm, in the region between the 3^rd^ and 5^th^ somites. This expression expands and shifts more posteriorly as the embryo grows: in embryos with 18 somites, expression extends up to the 13^th^ somite level and spans around 6 somites (Fig. 2F,G,Gii and data not shown). This expression of c*Hes6-1* in the endoderm resembles the location of pancreatic precursors in 1.5-day-old chick embryos, as determined by fate mapping [19], [20] and expression of the pancreatic markers *Nkx6-1,2,3, Nkx2.2* and *Pdx1* [21]. Since Notch signalling has been previously reported to be involved in pancreatic development [22], we also analysed the expression of other components of the Notch pathway in this region of the endoderm. We found that c*Hes6-2* is the only other *Hes* gene expressed in the same region, as we could not detect expression of c*Hes5-1*, c*Hes5-2*, c*Hes5-3*, c*Hairy1* or c*Hairy2* (Fig. 2H and data not shown). We also assessed the expression of Notch ligands and found that only c*Delta1* is expressed with a similar spatio-temporal pattern to c*Hes6-1* in the endoderm (Fig. 2I and data not shown).

To determine whether c*Hes6-1* expression in the embryo is under Notch control, we inhibited Notch signalling *in ovo* by treating embryos with a highly specific γ-secretase inhibitor (LY411575) [23] and assessed the effect on c*Hes6-1* expression. In the absence of Notch activity, c*Hes6-1* expression in the lateral mesoderm is strongly reduced (28/32 treated embryos, Fig. 2L), showing that c*Hes6-1* requires Notch signalling to be asymmetrically expressed in this region of the embryo. In the endoderm, on the contrary, inhibition of Notch activity leads to an increase in c*Hes6-1* expression (30/34 treated embryos, Fig. 2M), revealing that Notch signalling negatively regulates c*Hes6-1* expression in the prospective pancreas. This is consistent with a study showing that c*Hes6-1* is down-regulated in the developing pancreas of later stage embryos after overexpression of an active form of the Notch receptor [24]. Together, these results show that c*Hes6-1* is differently regulated in different regions of the developing chick embryo.

### c*Hes6-1* is expressed in post-mitotic cells in the outer ventricular zone of the spinal cord

**Figure 3:**
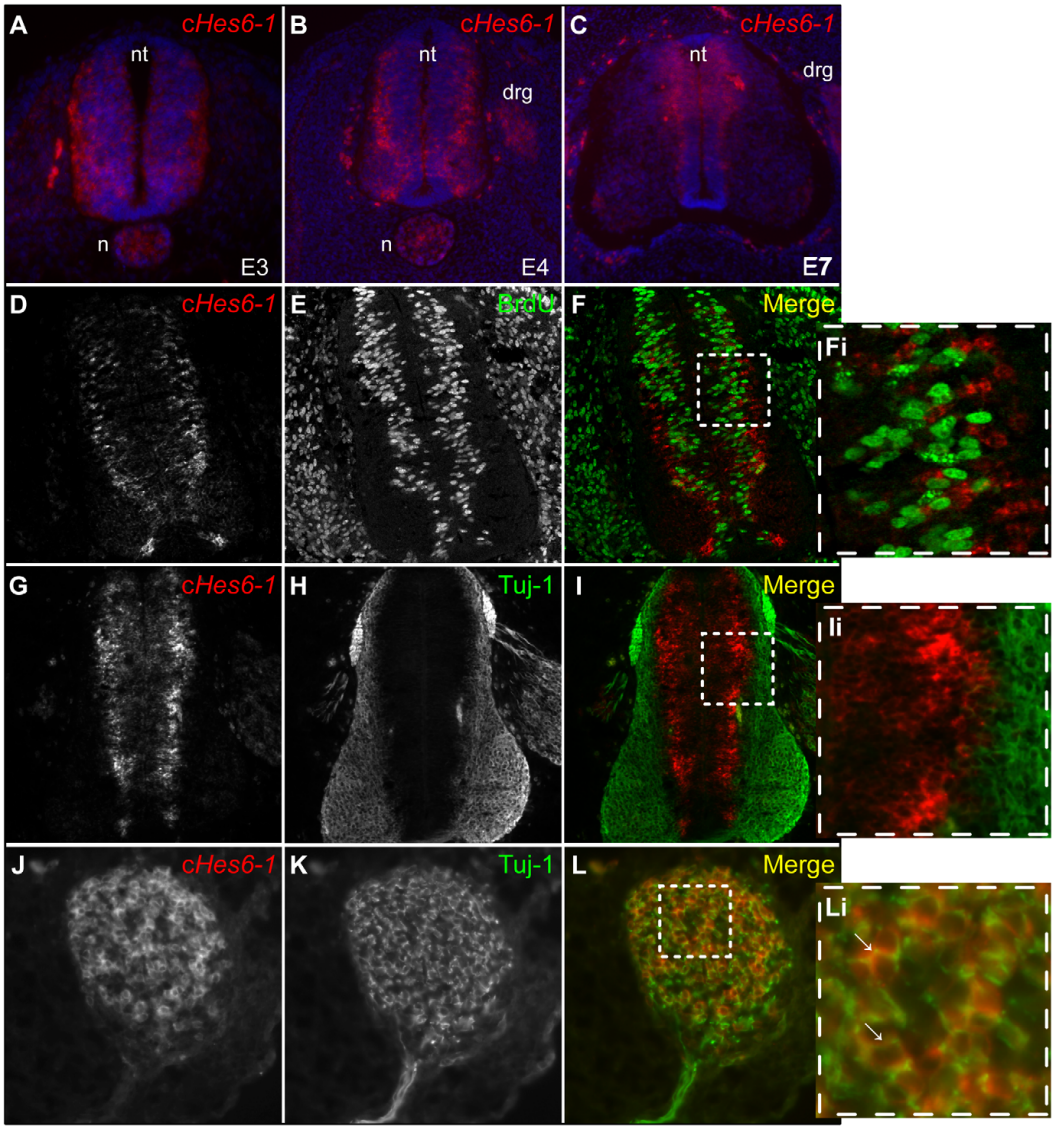
Expression of c*Hes6-1* in differentiating cells of the spinal cord. (A-C) Expression of c*Hes6-1* (red) in spinal cord sections of (A) E3, (B) E4 and (C) E7 embryos. Nuclei were counterstained with DAPI (blue). (D-Li) c*Hes6-1* (red) is expressed in post-mitotic cells of spinal cord of E4 embryos, as shown by the absence of BrdU incorporation (30 minute pulse (green)) in these cells (D-Fi). However, c*Hes6-1*-expressing cells are not fully differentiated, as demonstrated by absence of Tuj-1 co-labelling (green) (G-Ii). By contrast, in cranial ganglia c*Hes6-1* is expressed in Tuj-1^+^ differentiated neurons (J-Li). (Fi), (Ii), (Li) are magnifications of the selected areas in (F), (I), (L), respectively. Arrows identify cells where c*Hes6-1* expression coincides with Tuj-1. drg: dorsal root ganglion; n: notochord; nt: neural tube.

Analysis by whole-mount *in situ* hybridization shows that c*Hes6-1* is expressed throughout the developing nervous system and that the start of its expression coincides with the onset of neurogenesis at neural plate stages [25] (Fig. 2). To further examine the expression of c*Hes6-1* in the neural tube, we have performed *in situ* hybridizations in cryostat sections of E3-E7 embryos. While in E3 embryos, c*Hes6-1* expression is located throughout the spinal cord and expressing cells are mostly basal (Fig. 3A), in E4 embryos it is expressed in cells located at the border between the VZ and the mantle layer, across the whole dorso-ventral axis, (Fig. 3B). From E4 to E7, we observe c*Hes6-1* expression progressively disappearing from the ventral region, correlating with the completion of neurogenesis in this region (Fig. 3C).

To determine the proliferative status of c*Hes6-1*-expressing cells in the neural tube, we treated embryos *in ovo* with a pulse of Bromodeoxyuridine (BrdU) to mark cells in S-phase. Following immunohistochemistry for BrdU and *in situ* hybridization to detect c*Hes6-1* expression, we found that cells expressing c*Hes6-1* do not incorporate BrdU (Fig. 3D-Fi), showing that c*Hes6-1* is expressed in post-mitotic cells in the developing neural tube. However, double-labelling with Tuj-1, which marks early born neurons [26], does not show co-expression of c*Hes6-1* (Fig. 3G-Ii), indicating that this gene is expressed transiently during the initial phases of neuronal differentiation in the spinal cord. Nonetheless, a clear co-localization between c*Hes6-1* expression and Tuj-1 in dorsal root ganglia and cranial ganglia is observed (Fig. 3J-Li and data not shown), revealing a different timing for c*Hes6-1* expression in peripheral neurogenesis.

### c*Hes6-1* is part of the neuronal differentiation cascade of bHLH genes

**Figure 4:**
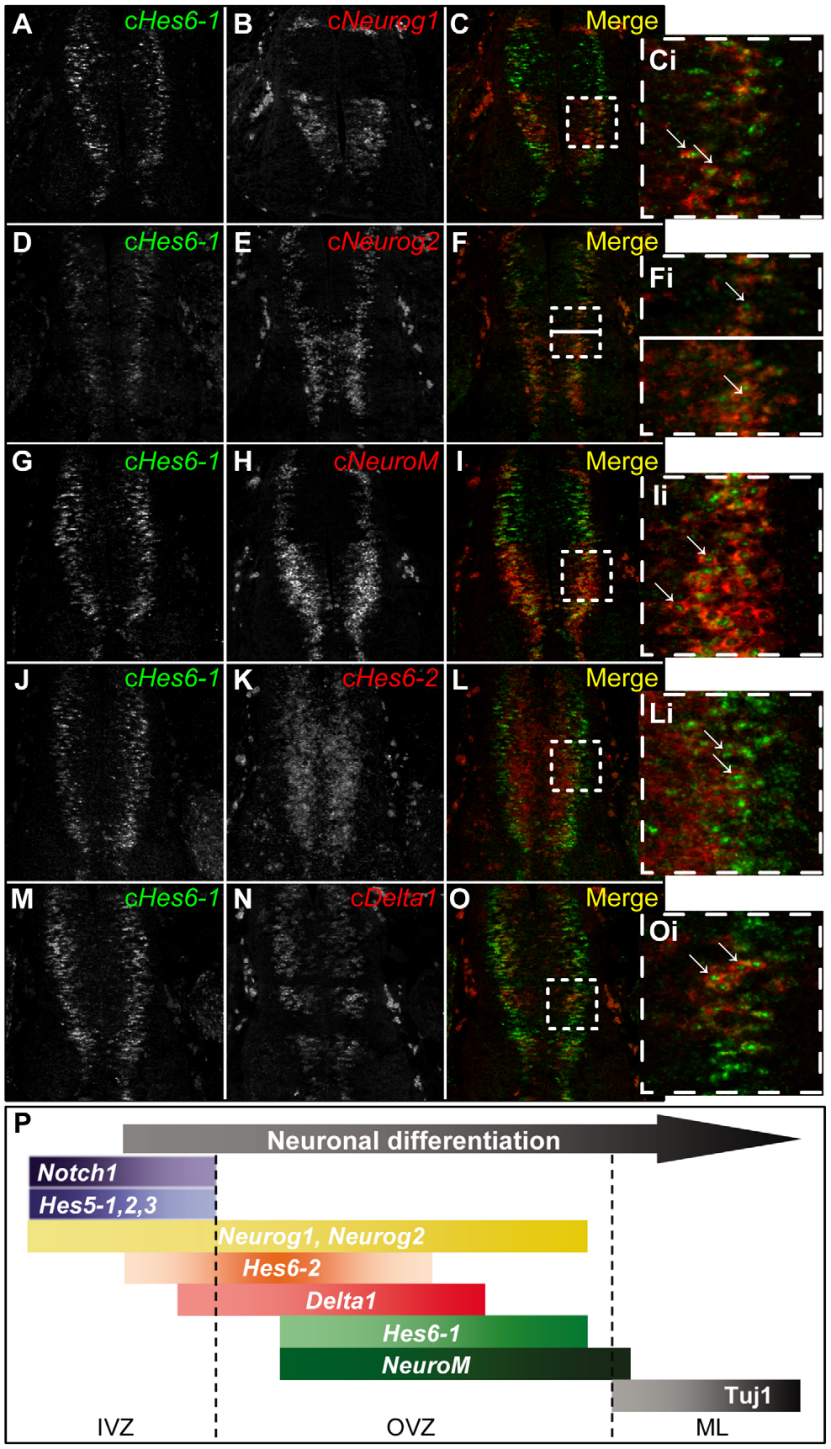
c*Hes6-1* expression relative to expression of various genes involved in neurogenesis. (A-Oi) Double *in situ* hybridization shows partial overlapping patterns and co-expression between c*Hes6-1* (green) and the following genes in spinal cord of E4 embryos: (A-Ci) c*Neurog1*, (D-Fi) c*Neurog2*, (G-Ii) c*NeuroM*, (J-Li) c*Hes6-2* and (M-Oi) c*Delta1* (red). (Ci), (Fi), (Ii), (Li), (Oi) are magnifications of the selected areas in (C), (F), (I), (L), (O), respectively. Arrows indicate cells where genes are co-expressed. Horizontal lines in (F) and (Fi) separate the dorsal and ventral domains of the spinal cord. (P) Neuronal differentiation is a step-wise process: differentiating neural progenitors exit the IVZ (inner ventricular zone) and sequentially activate neuronal differentiating genes during migration to the ML (mantle layer). c*Hes6-1* is transiently expressed in the OVZ (outer ventricular zone), where it is co-expressed with various genes involved in neurogenesis.

During vertebrate neurogenesis, a cascade of expression of proneural and neuronal differentiation genes encoding bHLH transcriptional regulators, such as Neurogenins, NEUROM and NEUROD1, regulate consecutive steps of differentiation (reviewed in [27], [28]–[30]). To relate the expression of c*Hes6-1* with the cascade of proneural bHLH expression, we performed double *in situ* hybridization with probes for c*Hes6-1* and various bHLH-encoding genes. We started with the proneural gene c*Neurog1* [31], which is mainly expressed in the ventral region of the developing spinal cord, with an additional dorsal stripe (dI2 [32]) (Fig. 4B). Analysis of *in situ* data revealed that cells in the VZ have different levels of c*Neurog1* expression and that cells with higher levels of c*Neurog1* are present more basally in the VZ, co-expressing c*Hes6-1* (Fig. 4A-Ci). We next analysed c*Neurog2* [33] expression, which in the ventral region is very similar to c*Neurog1*, with higher expressing cells also located more basally and co-expressing c*Hes6-1* (Fig. 4D-Fi). In the dorsal part of the neural tube, c*Neurog2*-expressing cells are uniformly located at the basal part of the VZ and show extensive co-expression of c*Hes6-1* (Fig. 4D-Fi). The finding that c*Hes6-1*-expressing cells show the highest levels of *Neurogenin* expression is in agreement with previous findings that show activation of HES6 expression by Neurogenins and cooperativity between both proteins to promote neuronal differentiation [8], [16].

Subsequently, we compared c*Hes6-1* expression to that of c*NeuroM*, which is expressed in differentiating neurons [34]. Double *in situ* hybridization shows co-expression of c*Hes6-1* and c*NeuroM* along the dorso-ventral axis, with cells co-expressing both genes located more apically than cells expressing only c*NeuroM* (Fig. 4G-Ii). This suggests that c*NeuroM* expression persists longer in differentiating neurons, after c*Hes6-1* expression is extinguished. Together, our results point to the following order of expression in differentiating neurons of the ventral spinal cord: c*Neurog1*/c*Neurog2* → c*Neurog1*/c*Neurog2*/c*NeuroM*/c*Hes6-1* → c*NeuroM* (Fig. 4P).

We have previously identified a circuitry of c*Hes5* and c*Hes6-2* activity during chick neurogenesis, with c*Hes6-2* functioning as a regulator of the three c*Hes5* genes [16]. Therefore, we compared the expression of c*Hes6-1* to that of c*Hes6-2* in these early steps of neuronal differentiation in the developing spinal cord. We found that the majority of c*Hes6-1*-expressing cells are located more basally than cells expressing c*Hes6-2*, although the two c*Hes6* genes are co-expressed in some cells (Fig. 4J-Li). This indicates that expression of c*Hes6-1* in newborn neurons occurs later than c*Hes6-2* expression. In relation to c*Delta1*, which is reported to be expressed after c*Hes6-2* [16], we found that this gene is expressed in cells located more apically that c*Hes6-1*-expressing cells, again with co-expression in some cells (Fig. 4M-Oi). Together, these results suggest the following sequence for the expression of these three genes in cells entering differentiation and initiating their migration out of the VZ: c*Hes6-2* → c*Delta1* → c*Hes6-1* (Fig. 4P).

### Regulation of c*Hes6-1* in the spinal cord

**Figure 5:**
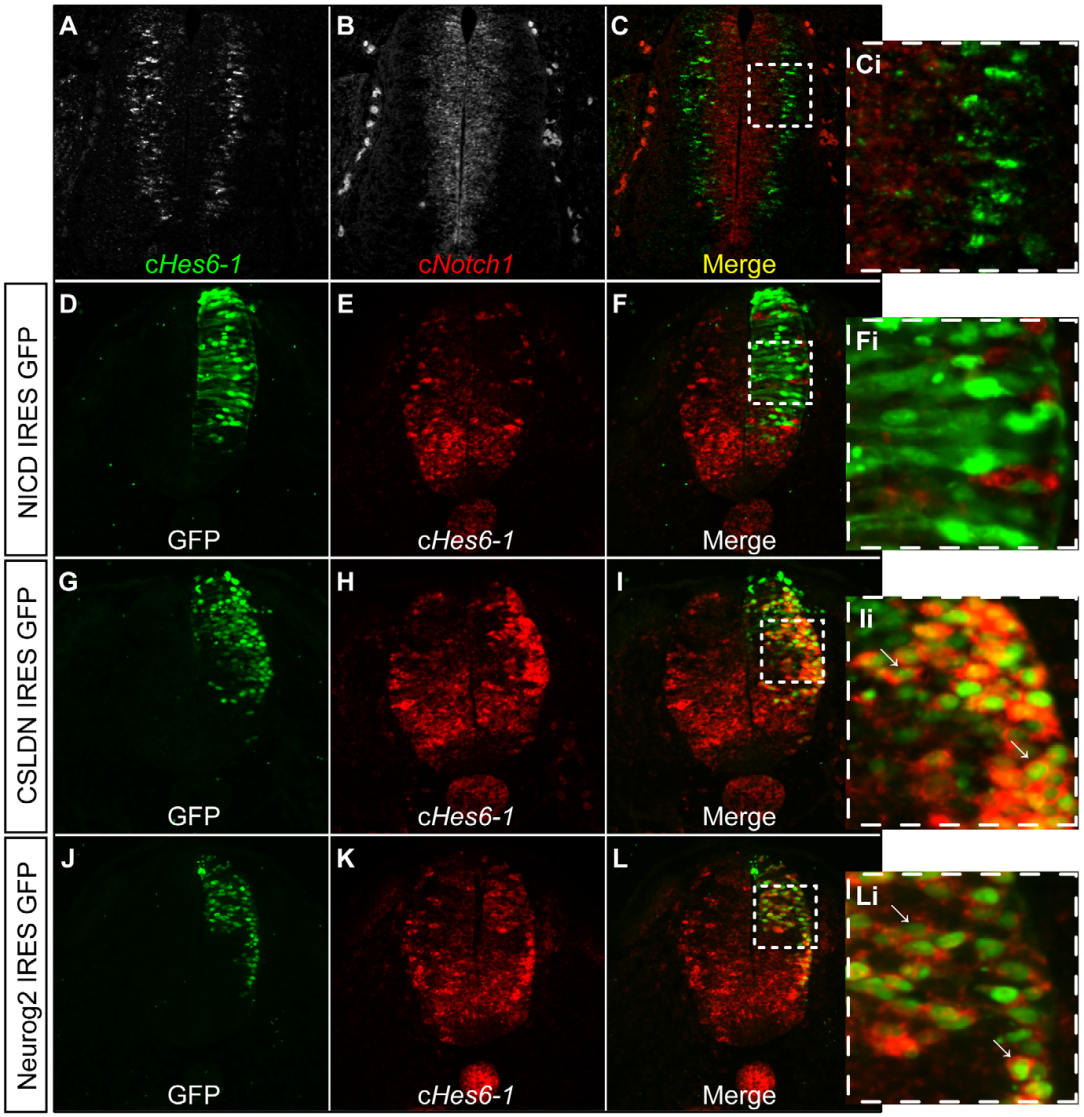
Regulation of c*Hes6-1* expression. (A-Ci) Double *in situ* hybridization for c*Hes6-1* (green) and c*Notch1* (red), showing no co-expression of the two genes in spinal cord of E4 embryos. (D-Li) c*Hes6-1* expression in the neural tube decreases after ectopic expression of NICD (D-Fi), and increases after ectopic expression of CSLDN (G-Ii) and NEUROG2 (J-Li). (Ci), (Fi), (Ii), (Li) are magnifications of the selected areas in (C), (F), (I), (L), respectively. Arrows pinpoint electroporated cells (GFP^+^) with increased expression.

The results described above show that c*Hes6-1* expression occurs in cells that have already committed to neuronal differentiation. Therefore, c*Hes6-1*-expressing cells should not present Notch activity, which is only present in progenitors to maintain their neurogenic potential. This is in agreement with non-overlapping expression domains of c*Hes6-1* and c*Notch1* in the developing spinal cord (Fig. 5A-Ci). Here, the two genes are transcribed in cells located in different regions of the neuroepithelium, c*Notch1* in apical progenitors at the ventricular zone and c*Hes6-1* in differentiating cells located at the border between the VZ and the mantle layer.

As c*Hes6-1* is expressed in differentiating neurons, interfering with neurogenesis should lead to alterations in its pattern of expression. To assess this, we examined the expression of c*Hes6-1* in the spinal cord of embryos where neurogenesis is blocked or accelerated, by manipulating Notch activity in the neuroepithelium. To block neurogenesis by increasing Notch activity, we electroporated chick embryos with a plasmid encoding a constitutively active form of the NOTCH1 receptor, NICD. Analysis of c*Hes6-1* expression in the spinal cord of these embryos shows that the number of cells expressing c*Hes6-1* is reduced when Notch is ectopically activated and neurogenesis is blocked (Fig. 5D-Fi). To accelerate neurogenesis by reducing Notch activity, we electroporated chick embryos with a plasmid encoding a dominant negative form of CSL (CSLDN). We also reduced Notch activity by treating embryos *in ovo* with the γ-secretase inhibitor LY411575 [23]. Analysis of c*Hes6-1* expression in the spinal cord of these embryos shows that the number of cells expressing c*Hes6-1* is increased when neurogenesis is accelerated due to Notch inactivation (Fig. 2N, 5G-Ii). The correlation of c*Hes6-1* expression levels with the rate of neurogenesis confirms that c*Hes6-1* is expressed in cells committed to neuronal differentiation, a process driven by the activity of proneural genes. Given our previous finding that c*Hes6-1* and the proneural gene *Neurog2* are co-expressed in various domains of the developing spinal cord (Fig. 4D-Fi), we next asked whether NEUROG2 can regulate c*Hes6-1* expression. To test this, a plasmid encoding NEUROG2 was electroporated into the chick neural tube. Upon NEUROG2 overexpression, we observe a cell-autonomous increase in c*Hes6-1* expression (Fig. 5J-Li), showing that c*Hes6-1* is expressed downstream of proneural genes during the process of neuronal differentiation in the developing spinal cord.

### cHES6-1 and cHES6-2 exert their function through different molecular mechanisms

**Figure 6:**
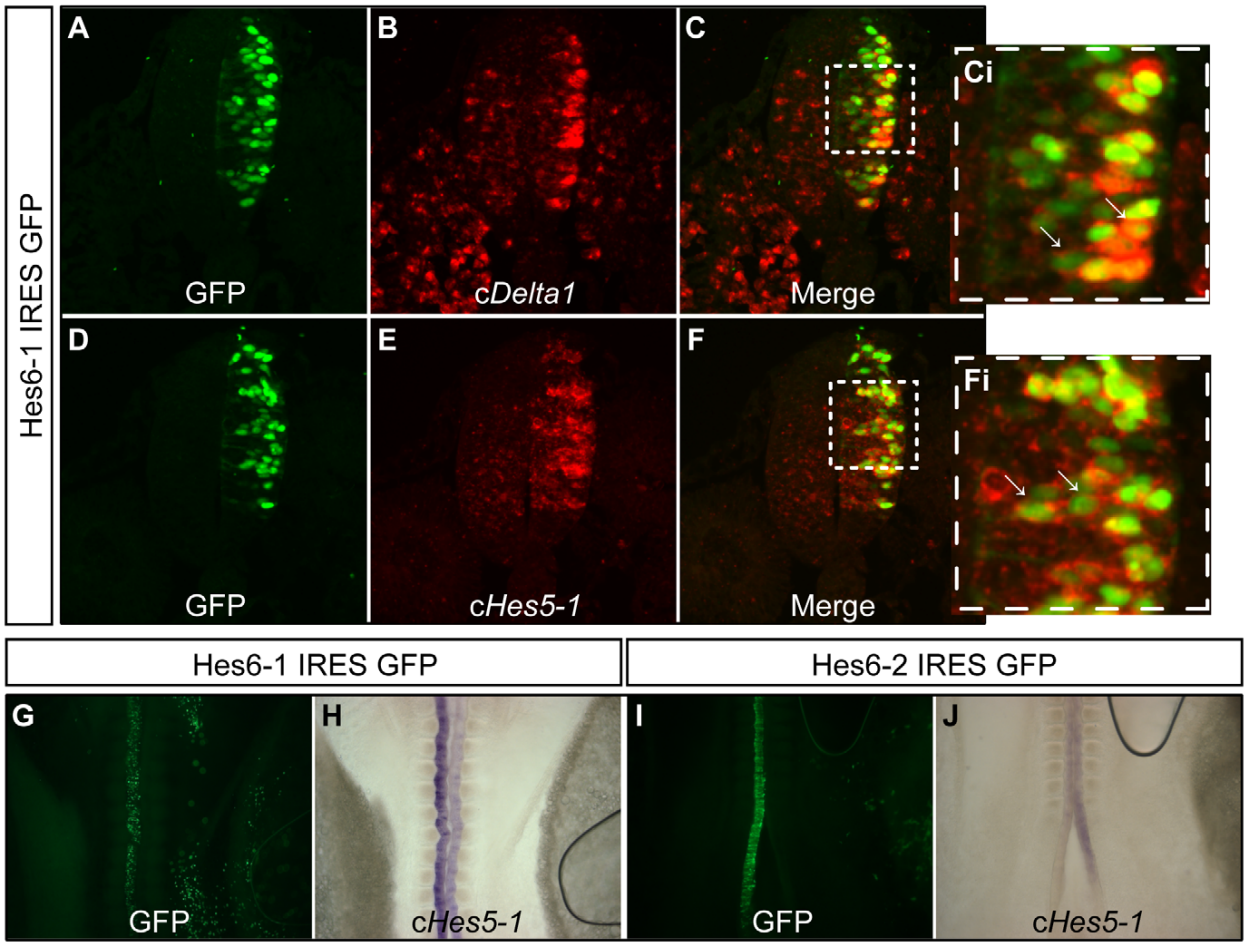
cHES6 overexpression phenotypes. (A-Fi) Overexpression of cHES6-1 causes up-regulation of (A-Ci) c*Delta1* and (D-Fi) c*Hes5-1*. (Ci), (Fi) are magnifications of the selected areas in (C) and (F), respectively. Arrows pinpoint electroporated cells (GFP^+^) with increased expression. (G-J) Whole-mount analysis of c*Hes5-1* expression in electroporated embryos shows that overexpression of cHES6-2 down-regulates c*Hes5-1* (I–J), contrasting with the up-regulation caused by cHES6-1 overexpression (G–H).

**Figure 7:**
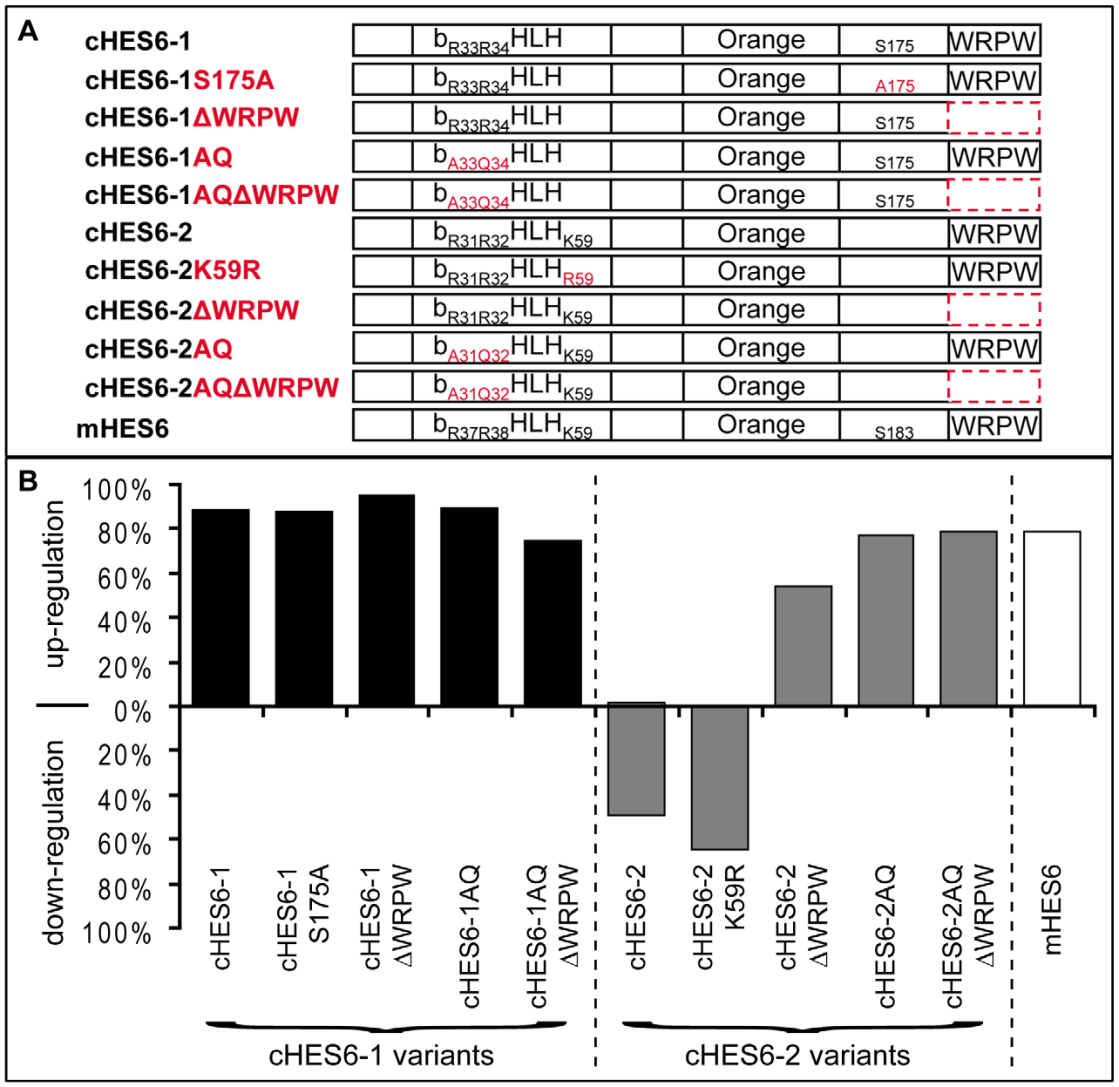
Phenotypical analysis of overexpressed cHES6 variants. (A) Schematic representation of various modified HES6 proteins overexpressed in the chick neural tube. Altered functional domains are depicted in red. (B) Percentage of embryos showing up- or down-regulation of c*Hes5-1* expression upon transfection with the different variants of cHES6-1 (black bars), cHES6-2 (grey) and mHES6 (white).

Our previous work with c*Hes6-2* revealed that this gene is active in differentiating neurons and serves to repress transcription of the three c*Hes5* genes encoding Notch effectors [16]. These are homologues of mouse *Hes5*, which has been shown to be the major target and effector of Notch signalling in the developing vertebrate nervous system [35], [36]. Therefore, c*Hes6-2* repression on the three chick *Hes5* genes helps to relieve differentiating neurons from Notch activity. Our finding that c*Hes6-1* is also expressed in differentiating neurons, starting later than c*Hes6-2*, but still overlapping with it (Fig. 4J-Li), suggests that c*Hes6-1* is also part of the mechanisms acting to cease Notch activity, promoting neuronal differentiation. To assess if c*Hes6-1* promotes neuronal differentiation, we ectopically expressed this gene in the embryonic chick neuroepithelium and analysed the expression of the early neuronal marker c*Delta1* [37]. Upon c*Hes6-1* overexpression, we observe a cell-autonomous up-regulation of c*Delta1* (80% of electroporated embryos, n = 44) (Fig. 6A-Ci), indicating that cHES6-1 promotes neuronal differentiation in a cell-autonomous fashion. This contrasts with the activity of cHES6-2, which represses c*Delta1* expression [16]. cHES6-2 is also a repressor of the c*Hes5* genes (Fig. 6I-J, 7B and [16]) and we observed that c*Hes6-1* overexpression leads also to a contrasting phenotype, causing cell-autonomous up-regulation of all three c*Hes5* genes (89% of electroporated embryos for c*Hes5-1*, n = 54) (Fig. 6D-H, 7B, and data not shown). Together, these results suggest that c*Hes6-1* and c*Hes6-2* act at different steps of the neuronal differentiation cascade and through different molecular mechanisms.

Comparison of the two subgroups of HES6 proteins reveals that a major structural difference is the size of the loop region in the bHLH domain, with the HES6-1 subgroup including proteins with shorter loops than HES6-2 proteins. The chick HES6-1 protein is particular in this aspect, as it contains the shortest loop (6 amino acids only) among all known vertebrate HES6 proteins (Fig. 1B). This unique structural feature might underlie the functional difference with cHES6-2 (which has a 10 amino acid loop) detected in our assay. Actually, previous work on the mouse counterpart of cHES6-1, mHES6, containing a 8 amino acid loop, revealed that this protein is unable to bind DNA due to its short loop, acting mainly through heterodimerization with other HES proteins to inhibit their DNA binding-dependent transcriptional repressor activity [8]–[11], [13].

Two other structural features might contribute to the different activities of cHES6 proteins. First, there is a specific serine residue in cHES6-1 (position 175), conserved in the HES6-1 subgroup but absent from HES6-2 proteins. Previous work on mHES6 has shown that phosphorylation of this serine (at position 183 of mHES6, within a putative PEST domain) by Casein Kinase 2 leads to proteolytic degradation of the mHES6-mHES1 heterodimer, thereby contributing to the assayed neurogenic ability of mHES6 [10]. The second feature is the presence of a conserved lysine in HES6-2 (K59 in cHES6-2), conserved in all mouse HES proteins with the exception of HES6, which contains an asparagine residue in similar position. This lysine was shown to be essential for the transcriptional repressive activity of mHES7, using *in vitro* transcription assays [17], and its absence in mHES6 was suggested to contribute also for the inability to repress transcription [17].

The short loop of cHES6-1, together with the presence of serine 175 and the absence of the lysine involved in transcriptional repression, suggest that cHES6-1 does not bind DNA to repress transcription, but rather interacts with other HES proteins to inactivate them. By contrast, the longer loop of cHES6-2, the absence of the serine, and the presence of lysine 59, all suggest that cHES6-2 functions by direct DNA binding and transcriptional repression. These observations raise the hypothesis that the functional differences between cHES6-1 and cHES6-2 in our assay arise from the different DNA-binding abilities of the two proteins, with cHES6-2 being able to bind DNA and acting as a transcriptional repressor of c*Hes5* genes, while cHES6-1 would instead work by forming inactive heterodimers with HES5 proteins. These latter proteins are known to bind DNA and repress their own transcription [16], so interference with their activity by ectopic cHES6-1 expression might underlie the observed up-regulation of c*Hes5* gene transcription.

To test this hypothesis, we engineered several variants of cHES6-1 and cHES6-2 proteins (Fig. 7A) and evaluated their activities in our electroporation assay, using the expression of c*Hes5-1* as a read-out. The first variants to be tested involved the substitution of lysine 59 of cHES6-2 by an arginine, as a similar mutation in lysine 55 of mHES7 abolished its ability to repress transcription [17]. Ectopic expression of cHES6-2K59R leads to down-regulation of c*Hes5-1* (65% of electroporated embryos, n = 20), a phenotype similar to that obtained upon overexpression of normal cHES6-2 protein (Fig. 7B). This result suggests that lysine 59 of cHES6-2 is not important for the function of this protein in our electroporation assay.

A variant of cHES6-1 was next generated where serine 175 is replaced by an alanine. A similar mutation in mHES6 was shown to impair the ability to target other HES binding partners for degradation [10]. Ectopic expression of cHES6-1S175A leads however to up-regulation of c*Hes5-1* (88% of electroporated embryos, n = 25), similar to overexpression of normal cHES6-1 (Fig. 7B), suggesting that serine 175 is not required for the activity of cHES6-1 in our electroporation assay.

We then tested whether removal of the C-terminal WRPW domain, which is known to be required for the transcriptional repressor activity of HES proteins by recruiting the TLE co-repressor, affects the function of chick HES6 proteins. We found that ectopic expression of cHES6-2ΔWRPW leads to up-regulation of c*Hes5-1* expression (54% of electroporated embryos, n = 13) (Fig. 7B), clearly contrasting with the activity of the non-modified protein that normally represses c*Hes5-1* transcription (49% of electroporated embryos, n = 63) (Fig. 6I–J, 7B). In turn, electroporation of cHES6-1ΔWRPW gives the same phenotype as normal cHES6-1, causing up-regulation of c*Hes5-1* transcription (95% of electroporated embryos, n = 19) (Fig. 7B). Together, these results show that the ability of cHES6-2 to repress transcription is compromised by its incapacity to recruit the co-repressor TLE, while cHES6-1 activity is not affected by the absence of the WRPW motif, supporting our hypothesis that cHES6-1 is not working as a DNA-bound transcriptional repressor in the developing spinal cord.

To directly test whether DNA-binding is involved in the differential activities of cHES6-1 and cHES6-2, we next electroporated variants of these proteins where two arginines from the C-terminus of the basic domain were replaced by uncharged amino acids (AQ), an alteration that has been shown to abolish the DNA-binding activity of several bHLH proteins [11], [13], [38]–[44]. A similar mutant of mHES6 (HES6AQ), in particular, was shown to lose the capacity to bind to Enhancer of Split E (ESE) motifs [11], [13], a specific type of E box recognized by the *Drosophila* Enhancer of Split proteins [45] that can also be recognized by mHES6 [11]–[13]. When cHES6-2AQ was electroporated in the embryonic neural tube, we observed an increase in c*Hes5-1* transcription (77% of electroporated embryos, n = 22), again contrasting with the repressive activity of the normal cHES6-2 protein. In turn, electroporation of cHES6-1AQ leads to an increase in c*Hes5-1* expression (89% of electroporated embryos, n = 19), similar to what we observe when misexpressing unmodified cHES6-1 (Fig. 7B).

In addition, we generated variants lacking both the WRPW domain and the ability to bind DNA (RR→AQ mutations), which we named cHES6-1AQΔWRPW and cHES6-2AQΔWRPW. Their electroporation lead to similar results to those obtained with the previous variants containing single modifications, causing up-regulation of c*Hes5-1* transcription (75% of electroporated embryos, n = 12, for cHES6-1AQΔWRPW and 78% of electroporated embryos, n = 37, for cHES6-2AQΔWRPW) (Fig. 7B).

Together, these results offer further support to the hypothesis that the two cHES6 proteins act through distinct molecular mechanisms to modulate c*Hes5* transcription in our assay, with cHES6-2 acting as a direct transcriptional repressor while cHES6-1 acts by preventing cHES5 proteins from repressing transcription of their own genes.

The proposed mechanism for cHES6-1 function is similar to that described for mouse HES6, which does not require DNA binding to inhibit HES1-mediated transcriptional repression, nor for the induction of neurogenesis, acting instead by forming inactive heterodimers with HES1 [8]–[11], [13]. We therefore tested mHES6 function in our electroporation assay and found that its overexpression in chick embryos causes the same phenotype as cHES6-1 (and opposite to cHES6-2), leading to similar up-regulation of c*Hes5-1* expression (79% of electroporated embryos, n = 14) (Fig. 7B).

Altogether, these results show that cHES6-1 does not require DNA binding nor the ability to recruit repressors for its function in regulating c*Hes5* expression, suggesting that it normally acts by directly sequestering the HES5 proteins. By contrast, DNA binding ability and recruitment of TLE are absolutely essential for cHES6-2 to function as a transcriptional repressor of c*Hes5* genes.

## Discussion

In this paper, we investigate a particular group of vertebrate *Hes* genes, named *Hes6*, and identify two subgroups that are present in zebrafish, medaka, *Xenopus*, and chick, which we named *Hes6-1* and *Hes6-2* (Fig. 1B). In contrast, only one *Hes6* gene is present in the mouse and human genomes, belonging to the *Hes6-1* subgroup. The two chick *Hes6* genes have been previously identified [16] but only the function of c*Hes6-2* has been investigated. We describe here the expression pattern and functional characterization of c*Hes6-1* during early embryonic development. Our results indicate that c*Hes6-1* shares a common function during neurogenesis with c*Hes6-2*, both contributing to relieve differentiating neurons from Notch activity, although through different molecular mechanisms.

### c*Hes6-1* expression and regulation

Characterization of c*Hes6-1* expression pattern reveals that this gene is active in all three germ layers, namely in the developing nervous system, pancreatic progenitors and, asymmetrically, in the left lateral mesoderm flanking the regressing node. This latter expression suggests that c*Hes6-1* participates in the mechanisms regulating the establishment of left-right asymmetry in the embryo. The signalling molecule NODAL is known to play a central role in these mechanisms, being asymmetrically expressed in the left lateral plate mesoderm, where it induces the expression of its downstream targets *Nkx3.2* and *Pitx2* to implement normal body asymmetry (reviewed in [46]). Expression of *Nodal* occurs sequentially in a wave-like fashion along the anterior-posterior axis, and reaches the posterior end of the embryo at the 5-somite stage, coinciding with the onset of expression of c*Hes6-1* in this region. In addition, when *Nodal* expression in the posterior lateral mesoderm is extinguished at late HH10, asymmetric expression of c*Hes6-1* also starts to fade out (data not shown). This correlation between the expression of *Nodal* and c*Hes6-1* suggests that this gene is a downstream target of Nodal signalling in the left mesoderm and might play a role in left-right asymmetry, in agreement with previous results showing that *Hes6* expression is downstream of Nodal signalling in *Xenopus* early gastrula embryos [47].

Notch signalling is also known to be an important player in L-R asymmetry, at the time when this asymmetry is first established around the Hensen's node (reviewed in [46]). However, it is not known if Notch also plays a role during the later stages when c*Hes6-1* is expressed. Our results show that asymmetric expression of c*Hes6-1* in the lateral mesoderm is dependent on Notch activity, thus revealing that this signalling pathway continues to be active during the process of generating L-R asymmetry in the embryo.

The transient and asymmetric expression of c*Hes6-1* raises the question of which asymmetric structures arise from the lateral mesoderm region expressing this gene. The position of c*Hes6-1*-expressing cells in the posterior mesoderm at HH10 suggests that these cells will end up flanking somites 18 and forward, as development proceeds. This region (between somites 18 and 21 of stage HH14 embryos) has been previously fate-mapped to be the main source of gonadal cells [48], which are known to develop differently between the two sides of the chick [49]–[51]. It is thus possible that the transient and asymmetric c*Hes6-1* expression in the lateral mesoderm might contribute to gonad asymmetry in chick development.

Early c*Hes6-1* expression also occurs in the ventral endoderm in a salt-and-pepper pattern, starting at 6-7-somite stage. The position of c*Hes6-1*-expressing cells suggests that they are pancreatic progenitors, although one of the first known markers of these cells, *Pdx1*, only starts to be expressed at the 9-10-somite stage [52]. However, pancreatic progenitors are known to have been already specified at the 6-somite stage [52], [53] and our results suggests that c*Hes6-1* is an early marker for these cells, before *Pdx1*. This agrees with the findings that mouse *Hes6* is also expressed in pancreatic cells and that its overexpression causes up-regulation of *Pdx1* expression [54].

We have found that c*Delta1* and c*Hes6-2* are also expressed in pancreatic progenitors at these early stages, suggesting that Notch signalling plays a role in the early stages of pancreatic cell fate specification. However, the finding that c*Hes6-1* expression is negatively regulated by Notch activity in prospective pancreatic cells indicates that this gene does not function as a Notch effector in the process. Instead, as suggested by studies of pancreatic cell differentiation at later stages [24], c*Hes6-1* is likely to be downstream of NEUROG3, participating in the cascade of bHLH proteins that regulate pancreatic differentiation. Since Notch signalling leads to repression of *Neurog3* expression during pancreas development [22], [24], this might explain the observed down-regulation of c*Hes6-1* expression by Notch activity.

In the developing spinal cord, c*Hes6-1* is transiently expressed during neuronal differentiation, in post-mitotic cells that co-express proneural transcription factors. Our expression analyses show that c*Hes6-1* and c*Hes6-2* are expressed at different steps of the neuronal differentiation cascade, with c*Hes6-2* being expressed before c*Delta1*, and c*Hes6-1* after, although co-expression of the two c*Hes6* genes is still observed in some cells. We further show that c*Hes6-1* expression is downstream of proneural transcription factors and is not a direct target of Notch signalling, being instead repressed by Notch activity, like in the prospective pancreas. Overall, our results suggest the existence of three consecutive stages in neuronal differentiation in the developing chick spinal cord: proneural proteins and cHES6-2 act first during the decision to commit to neurogenesis, followed by the expression of the Notch ligand cDelta1 to inhibit neighbouring progenitors from differentiating and, finally, by the expression of cHES6-1 and NEUROM to regulate subsequent differentiation steps (Fig. 4P).

### cHES6-1 and cHES6-2 have different mechanisms of action

To investigate the functional role of cHES6-1 during neurogenesis, we used gain-of-function studies in the chick embryo, by ectopically expressing the protein in the spinal cord neuroepithelium. Our findings reveal that ectopic expression of cHES6-1 promotes commitment to neuronal differentiation, as suggested by the cell-autonomous up-regulation of c*Delta1* expression. However, later markers for neuronal differentiation, like HuC/D and Tuj-1, are not induced by c*Hes6-1* overexpression (data not shown), suggesting that cells do not progress further into differentiation. This is likely due to the fact that transcription of c*Hes5* genes is also up-regulated by c*Hes6-1* expression, thereby repressing the neuronal differentiation cascade. Furthermore, the high levels of proneural gene expression required to trigger neuronal differentiation are not yet reached in all neural progenitors at the embryonic stages used for electroporation, a fact that might also explain why these cells are not able to terminally differentiate when electroporated with c*Hes6-1*.

The results of c*Hes6-1* misexpression are in striking contrast to the activities of the closely related gene c*Hes6-2* in the same assay, which causes down-regulation of both c*Delta1* and c*Hes5* expression. These functional differences are likely due to the unique structural features of each cHES6 protein, in particular the different sizes of the loop region within the bHLH domain, which is known to constrain the DNA-binding capacity of HES proteins [9]. Like its mouse counterpart mHES6, the chick HES6-1 protein contains a short loop in the bHLH region and might be also unable to bind DNA, functioning instead by sequestering anti-neurogenic HES proteins in transcriptionally inactive complexes, no longer able to repress their targets [8]–[11]. A conserved serine residue might also contribute to this function, as shown for mHES6, where it helps to target the mHES6/mHES1 heterodimer for degradation, after being phosphorylated [10]. In contrast, this serine is absent in cHES6-2, which contains a longer loop and might therefore function as a DNA-bound transcriptional repressor.

To test these hypotheses, we generated several variants of the two cHES6 proteins that predictably affect their DNA-binding capacity and/or their ability to repress transcription, and used these variants in the chick electroporation assay. Our results show that variants of cHES6-2 lacking the DNA binding ability, and/or the ability to recruit the co-repressor TLE, lose the capacity to repress c*Hes5-1* transcription, implying that cHES6-2 works normally as a DNA-bound transcriptional repressor. In contrast, similar variants of cHES6-1, where the ability to bind DNA and/or to recruit co-repressors is missing, are still able to up-regulate c*Hes5-1* transcription, like normal cHES6-1, showing that this protein does not require DNA binding nor transcriptional repression activity to function. This is further supported by our finding that overexpression of mHES6, a known protein titrator that does not require DNA binding nor the WRPW domain for its neurogenic activity [8]–[11], causes also up-regulation of c*Hes5-1* transcription, like cHES6-1. However, the conserved serine residue in cHES6-1 is not necessary for its capacity to up-regulate c*Hes5* expression, suggesting that phosphorylation and proteolytic degradation of cHES6-1/cHES5 heterodimers is not an absolute requirement for HES6-1 activity. Although a similarly mutated form of mHES6 (mHES6S183A) revealed no pro-neurogenic activity in a different assay using *in vitro* culture of cortical progenitors, proteolytic degradation of HES1 could still be detected in the presence of mHES6S183A [10]. In our assay, we didn't measure the neurogenic activity of cHES6-1 and the target is a different anti-neurogenic HES protein (HES5), so it is likely that serine phosphorylation of cHES6-1 could still be important in a different context where heterodimer formation with HES1 is the main mechanism to promote neurogenesis.

Together, our results support the hypothesis that, like its mouse counterpart mHES6 [9]–[11], [13], cHES6-1 works by binding to other HES proteins to inhibit their transcriptional repressive activity. In our assay, where most of the electroporated cells are c*Hes5*-expressing neural progenitors, the ectopic expression of cHES6-1 (or mHES6) would prevent cHES5 proteins from repressing transcription of their own genes, breaking this negative feedback loop and causing an increase in c*Hes5* transcription.

The related cHES6-2 protein, however, requires the presence of the WRPW domain and an intact DNA-binding region to effectively repress c*Hes5* transcription, strongly suggesting that this member of the HES6 family works as a classical DNA-bound transcriptional repressor. Thus, unlike the mouse, which contains a single HES6 gene, encoding a protein that works mainly by interfering with the anti-neurogenic activity of other HES proteins, the chick embryo contains two clustered HES6 genes that function through different but complementary mechanisms to regulate neuronal differentiation.

### What are the targets of cHES6-1?

**Figure 8:**
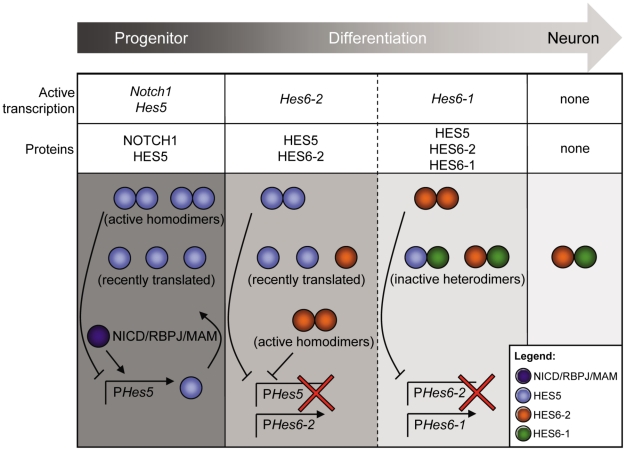
Model of cHES6-1 and cHES6-2 function during neurogenesis. During neuronal differentiation in the developing spinal cord, cHES6-1 and cHES6-2 act sequentially to relieve the cell from Notch signalling: cHES6-2 acts first to repress the transcription of c*Hes5* genes and cHES6-1 subsequently sequesters and inactivates cHES5 and cHES6-2 proteins.

During the normal process of neurogenesis, we have shown that c*Hes6-1* is expressed in differentiating neurons, which normally do not express c*Notch1* or any c*Hes5* gene. However, it is possible that cHES5 proteins perdure in cells committing to differentiation, even if their mRNAs cannot be detected anymore, and cHES6-1 would ensure that any remaining cHES5 activity is blocked. Another possible target of cHES6-1 is cHES6-2, as the encoding mRNAs can be detected in the same cells during neuronal differentiation (Fig. 4J-Li). We have shown previously that cHES6-2 helps to release the differentiating neuron from Notch signalling, although its activity in the absence of proneural proteins could also lead to a block in neurogenesis [16]. It is therefore important to inhibit cHES6-2 for neuronal differentiation to proceed and this function might be accomplished by cHES6-1, which is expressed later than cHES6-2 in differentiating neurons (Fig. 4J-Li). We thus suggest that the two c*Hes6* genes are part of a mechanism that functions to ensure that Notch signalling is completely turned off when neuroepithelial cells enter differentiation (Fig. 8). During this process, c*Hes6-2* acts first as a transcriptional repressor of the progenitor program by repressing transcription of the c*Hes5* genes encoding Notch effectors. This might lead to a major increase in proneural gene expression which reinforces c*Hes6-2* transcription and activates c*Hes6-1*. Finally, cHES6-2 activity is turned off by the activity of cHES6-1 and also because it represses transcription of its own gene [16]. Together, the two cHES6 proteins progressively and effectively shut down the Notch-mediated progenitor program, making sure that neuronal differentiation can proceed.

## Materials and Methods

### Embryo electroporation

For NICD, CSL and NEUROG2 expressing plasmids, super-coiled DNA was injected into neural tubes of chicken embryos staged HH11-HH13 or HH16-HH17 at a concentration of 2 µg/µl in PBS and fast green was used for contrast. Platinum electrodes (Nepagene CUY613P5), distanced 4 mm apart, were placed parallel to the neural tube under the embryo. Using an Electro Square Porator™ ECM830 (BTX), we applied 4 pulses of 35 V for 50 ms. Embryos were incubated for 16 h or 24 h and then harvested.

For each construct, a minimum of 4 electroporated embryos were analysed by *in situ* hybridization and immunofluorescence. The results obtained after 16 h or 24 h were identical.

For the various HES6 plasmids, super-coiled DNA was injected into neural tubes of chicken embryos staged HH8-HH11 at a concentration of 1 µg/µl. Gold plated electrodes (BTX Genetrode 512), distanced 4 mm apart, were placed parallel to the neural tube on the top of the embryo. Using an Electro Square Porator™ ECM830 (BTX), we applied 4 pulses of 25 V for 50 ms. Embryos were incubated for 6 h and then harvested.

### Plasmids

The expression plasmids encoding NICD, CSLDN, NEUROG2 and cHES6-2 have been already described [16]. Plasmid constructs to express the various HES6 proteins were generated in pCIG [55]. In all expression plasmids, IRES∶GFP is present to allow identification of electroporated cells. All clones were screened for the correct orientation of the insert and were checked by sequencing. Primers and cloning details are available upon request.

### LY411575 treatment

To chemically inhibit Notch activity *in ovo*, 100 µl of the γ-secretase inhibitor LY411575, at 10 µM in PBS, were injected under 3–8 somite staged embryos, in three independent experiments. These embryos were harvested 7 h later. Control was done with similar injections of PBS alone (17 embryos for the asymmetry study, 23 for the pancreatic progenitors and 23 for the neural tube). After 7 h of treatment, control embryos had an average increase of 5 somites, as expected. In contrast, LY411575-treated embryos only had an average increase of 3 somites. This significant difference in the rate of somite formation between control and LY411575 treated embryos (*t*-test; p-value<0.001), suggests that the drug was effective in inhibiting Notch signalling during somitogenesis.

### *In situ* hybridization and immunofluorescence

Chicken embryos were collected and fixed in 4% paraformaldehyde in PBS overnight at 4°C. Whole-mount *in situ* hybridizations were done as described [37], with modifications. For hybridization on cryostat sections, fixed embryos were cryoprotected in 30% sucrose in PBS, embedded in a solution containing 7.5% gelatine and 15% sucrose in PBS, and cryosectioned (12 µm). Hybridization on cryostat sections was done as previously described [56], with modifications. Double *in situ* hybridization on cryostat sections was done with Digoxigenin (Dig)- and Fluorescein (Fluo)-labelled RNA probes. The Dig-labelled probe was first detected with AP-conjugated anti-DIG antibody (1∶2000, Roche) and developed with Fast-Red substract (Roche). After washing in PBS, sections were blocked and incubated with HRP-conjugated anti-Fluo antibody (1∶1000, Roche), followed by FITC-Tyramide amplification, as recommended by the manufacturer (TSA™-Plus Fluorescein System Kit, Perkin-Elmer).

For the analysis of HES6 electroporations in the neural tube, c*Delta1* and c*Hes5-1* riboprobes have been synthesized from specific PCR fragments, to avoid any plasmid regions in the probes and eliminate regions of putative cross reactivity between the probes and transgenes.

Electroporated cells were visualized after *in situ* hybridization by immunohistochemistry using a rabbit polyclonal antibody against GFP (1∶500, AbCam). For BrdU treatment, 100 µL (12.5 mg/mL) were dropped onto E4 chick embryos, which were harvested and fixed 30 minutes later. Antigen retrieval for BrdU-treated embryos was done using HCl 2N for 30 min at 37°C. Primary antibodies used were mouse anti-BrdU (1∶1000, Sigma) and mouse anti-Tuj-1 (1∶500, Covance). Detailed protocols are available upon request. Photos were taken using the stereoscope LeicaZ6APO with a DFC490 camera attached, fluorescent microscopes Leica DMR or Leica DM5000B with cameras DC350F or DC500 attached, and the confocal Carl Zeiss 510 Meta.
